# Co-Occurrence of SARS-CoV-2 Infection and Inactivated SARS-CoV-2 Vaccination among Healthcare Workers

**DOI:** 10.1155/2021/3006251

**Published:** 2021-11-03

**Authors:** Cucunawangsih Cucunawangsih, Ratna Sari Wijaya, Nata Pratama Hardjo Lugito, Ivet Suriapranata

**Affiliations:** Faculty of Medicine, Pelita Harapan University, Tangerang, Indonesia

## Abstract

The presented cases describe the concurrent SARS-CoV-2 infection and inactivated SARS-CoV-2 vaccination among eight healthcare workers (HCWs). These cases highlighted the importance of broad hospital screening during the COVID-19 vaccination campaign. Further study regarding the durability of antibody response induced by infection and first-dose vaccination is required to determine the appropriate time for giving a second dose of inactivated SARS-CoV-2 vaccine among these cases.

## 1. Introduction

Since the first coronavirus disease 2019 (COVID-19) case caused by severe acute respiratory syndrome coronavirus 2 (SARS-CoV-2) was detected in China in December 2019, the disease has spread rapidly worldwide. Indonesia is one of the Southeast Asian countries with a high number of confirmed and active COVID-19 cases [1]. Although preventive measures such as physical distancing, quarantine, and isolation effectively reduced the number of people becoming infected, the risk of SARS-CoV-2 infection persisted in the population without immunity against SARS-CoV-2. Therefore, the availability of the COVID-19 vaccine is essential to induce immunity and protect the population from SARS-CoV-2 infection.

In Indonesia, the COVID-19 vaccination campaign using inactivated SARS-CoV-2 vaccine (CoronaVac, Sinovac Life Sciences) started at the end of January 2021, initially prioritizing healthcare workers (HCWs). As CoronaVac is an inactivated vaccine containing a whole virus structure [2], vaccinated individuals would be expected to elicit antibodies against many SARS-CoV-2 antigens, such as antispike (anti-S) and antinucleocapsid (anti-N). The remarkable increase of neutralizing antibodies, spike-specific immunoglobulin G (IgG), and receptor-binding domain- (RBD-) specific IgG occurred on day 14 after the second dose of vaccination [3]. Although the most common adverse reaction was injection site pain, systemic reactions such as fever, fatigue, cough, myalgia, and headache have been reported after each injection [3, 4].

The following presented cases, showing the co-occurrence of the first time use of CoronaVac with positive SARS-CoV-2 RNA among HCWs, are important due to it raising several considerations related to (1) the possibility for misinterpretation of COVID-19 symptoms with the systemic adverse reaction of vaccine, (2) the possibility of a false-positive RT-PCR result caused by the vaccine, and (3) the safety and the durability of the immune response during the coincidental events of vaccination and SARS-CoV-2 infection.

## 2. Case Presentation

This is an eight-case series of HCWs who received the first dose of inactivated SARS-CoV-2 vaccine (CoronaVac) at the Siloam Teaching Hospital (Indonesia) on January 26, 2021. The time elapsed between the first dose of vaccination and the onset of symptoms ranged from 4 to 9 days (median time 6 days). HCWs were confirmed for SARS-CoV-2 detection by RT-PCR in nasopharyngeal swab samples collected between 2 and 7 days after the onset of the symptoms (Table 1).

The mean age of vaccinated HCWs diagnosed with COVID-19 was 31.1 years (±6.8 years), and 3 (37%) were male. Most of the vaccinated and infected HCWs work as a nurse (63%). Five (63%) HCWs were infected in the community setting, and three (37%) were from a healthcare setting, who might have acquired it through contact with SARS-CoV-2-positive patients or coworkers.

**Figure 1:**
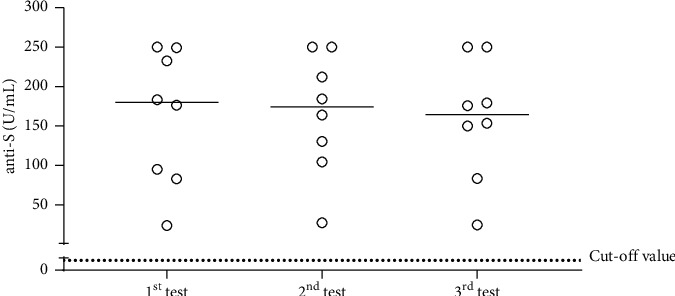
Serial measurement of anti-S antibodies' response among infected HCWs after acquiring the first-dose inactivated SARS-CoV-2 vaccination. Antibodies against RBD of the S protein (anti-S) concentration among eight HCWs who get SARS-CoV-2 infection after receiving the first dose of the inactivated SARS-CoV-2 vaccination are shown. The anti-S antibody concentration was measured on days 30, 60, and 90 after the RT-PCR positive result. The black dotted horizontal line represents the cutoff value (0.8) suggested by the manufacturer for positive interpretation of antibodies to SARS-CoV-2.

Among these 8 HCWs, 6 (75%) were tested because they had COVID-19 symptoms. The most common COVID-19 symptoms were fever (75%), cough (25%), and headache (25%). Two (25%) asymptomatic COVID-19 HCWs were identified as a part of postexposure and regular hospital screening. Only one subject (HCW7) had preexisting medical conditions. The mean cycle threshold (Ct) values of the N gene and ORF1ab gene were 24.7 (±7.1) and 25.9 (±7.8), respectively. The total antibodies against S1-RBD protein (anti-S) were measured using Elecys anti-SARS-CoV-2 S assay and analyzed on the Cobas e601 platform (Roche Diagnostics, Switzerland). According to manufacturer's guidelines, sample values ≥ 0.8 U/mL were interpreted as positive for anti-S antibodies. The antibody measurement was performed at three time points: on days 30, 60, and 90 after the positive RT-PCR test (Figure 1). The result showed the seroconversion observed in all HCWs on day 30 after the positive RT-PCR test, and the anti-S antibody concentration continued to be stably detected until day 90. No significant difference of anti-S antibody concentration on days 30, 60, and 90 after the RT-PCR positive test was observed (*p* > 0.05, Figure 1). The clinical outcomes of vaccinated HCWs with COVID-19 were favourable in all cases, with no hospitalization and no mortality observed among study cases.

## 3. Discussion

Compared to the general population, HCWs have a higher risk of SARS-CoV-2 infection, and the infected HCWs possess a greater risk of transmitting and spreading the infection in hospital and community settings [5]. Therefore, HCWs were prioritized to receive the vaccine in the initial COVID-19 vaccination program in Indonesia. However, in the situation where the vaccination program coincides with the high daily confirmed cases of COVID-19 like in Indonesia, more cases like described above will be expected.

Fever, the most prevalent symptom observed among COVID-19 HCWs in this study, is the common systemic reaction after vaccination with an inactivated COVID-19 vaccine [3, 4]. Consequently, the misdiagnosis of COVID-19 with vaccination side effects is likely to occur. Considering HCWs represent a high-risk group for SARS-CoV-2 exposure, the presence of any symptoms after vaccination cannot be ignored as a vaccination side effect until a further diagnostic test can rule out the COVID-19 diagnosis. In addition, most HCWs acquired the infection through community settings. This result underscores the importance of the high-level awareness of reported symptoms from vaccinated HCWs, particularly in a region where daily confirmed COVID-19 cases are still high. Furthermore, two infected HCWs did not experience any symptoms, which can be a potential transmission source in hospital and community settings [6]. Altogether, these results imply that the hospital needs to be vigilant and introduce regular COVID-19 testing for all HCWs.

Although the false-positive RT-PCR result after vaccination has been reported after administrated intranasal live attenuated influenza vaccine (LAIV) [7, 8], the positive RT-PCR among vaccinated HCWs is possibly not due to the COVID-19 vaccine. The vaccine administration of LAIV and specimen collection for testing were in the same site, resulting in the possibility of positive detection by RT-PCR [7, 8]. In contrast, the COVID-19 vaccine was administrated intramuscularly into deltoid muscle, in which the protein antigen is taken up by antigen-presenting cells (APCs) and then trafficked to the lymph node for adaptive immune cell activation. As a result, it is possibly unlikely to find the trace of the vaccine component in the specimen collection site for RT-PCR.

Furthermore, we observed the favourable clinical outcomes of COVID-19 among vaccinated HCWs, as all of them did not need hospitalization and no one succumbed. This result may indicate that the coincidental inactivated COVID-19 vaccine administration, and SARS-CoV-2 infection seemed to be well tolerated and not causing the overregulation of the immune system. The seroconversion of anti-S was observed in all HCWs after 30 days of the RT-PCR test. Anti-S concentration was heterogeneous among HCWs (range 23.8 to 250, median 179.8), as has been widely described [9, 10]. The antibody was relatively stable until 90 days after the RT-PCR test. Considering that all presented HCW cases in this study were not eligible for acquiring a second dose of the inactivated SARS-CoV-2 vaccine, the longer-term follow-up is required to investigate the durability of observed anti-S antibodies beyond this time point in order to decide the appropriate time for giving a second dose of COVID-19 vaccine among these study cases.

## Figures and Tables

**Table 1:** COVID-19 cases among first-dose vaccinated healthcare workers (HCWs) at Siloam Teaching Hospital, Indonesia, January 26–February 9, 2021.

No.	Age	Sex	Occupation	Probable source of contamination	Symptoms	Comorbidities	Date of the 1^st^ vaccine	Date of RT-PCR (+)	Date of symptoms	Indication for testing	Day of symptom onset^a^	Number of days from symptom onset to testing	Ct values target 1 (N)	Ct values target 2 (ORF1ab)	Hospitalization	Death

1	25	F	Nurse	Community	Fever, headache	None	01 February 2021	14 February 2021	10 February 2021	Symptoms	9	4	25.3	22.1	No	No

2	26	F	Staff	Community	Fever	None	29 January 2021	08 February 2021	02 February 2021	Symptoms	4	6	Neg	33.7	No	No

3	40	F	Nurse	Community	Fever, headache	None	04 February 2021	12 February 2021	10 February 2021	Symptoms	6	2	31.1	31.1	No	No

4	29	F	Nurse	Healthcare setting	Fever, cough	None	03 February 2021	17 February 2021	10 February 2021	Symptoms	7	7	16.1	16.5	No	No

5	31	M	Housekeeper	Community	Fever	None	05 February 2021	14 February 2021	10 February 2021	Symptoms	5	4	18.3	18.5	No	No

6	34	M	Staff	Healthcare setting	NA	None	01 February 2021	03 February 2021	Asymp	Exposure	NA	NA	31	31.5	No	No

7	41	F	Nurse	Healthcare setting	Fever, cough	Diabetes	01 February 2021	09 February 2021	07 February 2021	Symptoms	6	2	32.7	35.5	No	No

8	23	M	Nurse	Community	NA	None	02 February 2021	09 February 2021	Asymp	Exposure	NA	NA	18.1	18.5	No	No

^a^Considering the day of the first vaccination as day 0. NA, not applicable; Ct, cycle threshold; Asymp, asymptomatic; RT-PCR, reverse-transcriptase polymerase chain reaction; Neg, negative.

## Data Availability

The data used to support the findings of this study are included within the article.
